# Challenging students to formulate written questions: a randomized controlled trial to assess learning effects

**DOI:** 10.1186/s12909-015-0336-z

**Published:** 2015-03-21

**Authors:** Marleen Olde Bekkink, A R T Rogier Donders, Jan G Kooloos, Rob MW de Waal, Dirk J Ruiter

**Affiliations:** 1 Department of Anatomy, Radboud University Nijmegen Medical Centre, P.O. Box 9101, 6500 HB Nijmegen, The Netherlands; 2 Department of Internal Medicine, Radboud University Nijmegen Medical Centre, Nijmegen, The Netherlands; 3 Department for Health Evidence, Radboud University Nijmegen Medical Centre, Nijmegen, The Netherlands; 4 Department of Pathology, Radboud University Nijmegen Medical Centre, Nijmegen, The Netherlands

**Keywords:** Small-group teaching and learning, Written questions, Small-group dialogue, Student performance, Gender differences

## Abstract

**Background:**

Underutilization of dialogue among students during small-group work is a threat to active meaningful learning. To encourage small-group learning, we challenged students to generate written questions during a small-group work session. As gender differences have been shown to affect learning, these were also inventoried.

**Methods:**

Prospective randomized study during a bachelor General Pathology course including 459 (bio) medical students, 315 females and 144 males. The intervention was to individually generate an extra written question on disease mechanisms, followed by a selection, by each student group, of the two questions considered to be most relevant. These selected questions were open for discussion during the subsequent interactive lecture. Outcome measure was the score on tumour pathology (range 1–10) on the course examination; the effect of gender was assessed.

**Results:**

The mean score per student was 7.2 (intervention) and 6.9 (control; p = 0.22). Male students in the intervention group scored 0.5 point higher than controls (p = 0.05). In female students, this was only 0.1 point higher (p = 0.75).

**Conclusions:**

Formulating and prioritizing an extra written question during small-group work seems to exert a positive learning effect on male students. This is an interesting approach to improve learning in male students, as they generally tend to perform less well than their female colleagues.

## Background

Active meaningful learning is supposed to play a central role in medical education, especially during small-group work (SGW) sessions and interactive lectures. This type of learning is driven by continuing dialogue among students, and between students and tutors, creating a constructive educational environment that enhances conceptual understanding based on the constructivist theory of learning [1]. During group discussions, students learn effectively, and knowledge is retained longer when they are able to engage in active learning [2,3]. The quality of such small-group dialogue is crucial to the progress of a student with respect to all aspects of learning, i.e. knowledge, metacognitive skills and attitude [4]. As student-centered learning is moving towards participatory education [5,6], endowing students with the role of co-creator of their education, it is a pre-requisite that students’ input in the small-group dialogue increases. In earlier studies, students have indicated that group interaction and active student participation as well as the opportunity to ask questions are essential components of effective SGW [7]. However, based on our experience and that of others [8-11], it is apparent that underutilization of this dialogue during SGW occurs during our medical and biomedical science educational programmes, both quantitatively and qualitatively. This underutilization seems to be due to time pressure, lack of motivation and poor preparation. As these are regarded to hamper active meaningful learning, strategies that could improve the small-group dialogue are considered.

Students’ ability to generate and formulate written questions is a key skill required for effective small-group learning [12]. Raising written questions has four important educational aspects: (1) it is a measure of curiosity which is a natural driver of learning [13]; (2) it stimulates active participation in the learning and instructional process [14,15]; (3) it stimulates critical thinking and thereby academic performance [16]; and (4) it is informative about a student’s progress, and an obvious source of feedback by the tutor. By generating and formulating written questions, students are stimulated to reflect on their learning progress and start to develop metacognitive capacity [17], an important competency for medical doctors and biomedical professionals. Therefore, it is interesting to explore whether challenging students to generate written questions and prioritize them during a SGW session of an ongoing (bio) medical course would be effective as a strategy to improve learning performance. Students were invited to formulate their questions in writing, instead of only verbalizing them, in order to increase stringency. The rationale for asking students to prioritize the questions is twofold: to stimulate students to verbalize why they think their question is important; and, subsequently, to elicit group discussion by feeding the dialogue among students. Group discussion of questions could stimulate students to elaborate on their learning [18]. As the motivation of female (bio) medical students for learning may be of a different nature (intrinsic versus extrinsic) than that of their male peers [19], gender differences in the effect of the intervention might be present as well. Extrinsic motivation (e.g. summative tests, status, expected income) is more prominent among male students. Females generally have higher intrinsic motivation, implicating that they are genuinely interested and curious to learn more about the topic.

Based on these considerations, this study was executed to determine: (1) if an intervention directed at formulating and discussing an extra written question by the students during SGW would have a positive learning effect, i.e. an effect on the formal examination score; and (2) if gender influences the effect of this intervention. This was done by means of a prospective randomized study.

## Methods

### Participants and setting

The study was conducted with (bio) medical students at the Radboud University Medical Centre, Nijmegen, the Netherlands. Participants were 315 female and 144 male students who were undertaking a second-year Bachelor course on General Pathology. The study discipline ratio of Medicine to Biomedical Sciences of the participants was 3:1. A learner outcome-oriented curriculum consisting of consecutive courses was provided in which each course lasted four weeks. The successive topics of the course on General Pathology were: (1) principles of diagnosis and cellular damage; (2) inflammation and repair; (3) circulatory disorders; and (4) tumour pathology (pathogenesis and progression). Each topic had a consistent sequence of educational activities: lecture; task-driven directed self-study in preparation for the subsequent small group work; small group work (obligatory); practical course (obligatory); interactive lecture; and non-directed self-study. The study was executed during the SGW session on the topic of tumour pathology (2 hours) during the fourth week. These sessions involved groups of 12–15 students. On the final day of the course, the students were subjected to a formal examination on all four topics.

### Intervention and procedure

**Figure 1:**
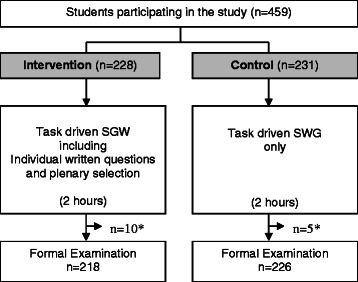
**Study design including the intervention and control arms.** *Number of students excluded because they did not participate in the formal examination (n = 15).

At the start of the SGW, the tutor invited the students to formulate an extra written question related to the topic of tumour pathology. It was stressed that this should be a deepening question on disease mechanisms and not mere factual knowledge. The students were instructed to think about the extra question during the SGW. At the end of the SGW, the students individually wrote down at least one of their questions, and immediately afterwards the two most relevant questions per SGW were selected after a short plenary discussion. The intervention (writing the question followed by the plenary discussion) lasted for a maximum of 10 minutes. Participation was on a voluntary basis and written informed consent was obtained. The students were invited, on a voluntary basis, to discuss the selected questions during the subsequent interactive lecture that was held the next day. Whether or not the students actually did raise the questions during the interactive lecture was not controlled for. In the control groups, the usual task-driven discussions on tumour pathology lasted until the end of the SGW session. The total exposure time to the topic was similar in the intervention and control groups (see Figure 1).

### Randomization

The participants were randomly assigned to one of two arms with equal numbers of SGW groups. Allocation of intervention occurred at the SGW group level. Gender rates were calculated for each group. A minimization procedure according to Pocock and Simon [20] and Borm et al. [21] was used to obtain optimal balance on the factors gender, study discipline and tutor, as they may influence learning behaviour and learning efficacy [19].

### Outcome measure

Outcome measure was the subscore on the course examination multiple-choice questions on tumour pathology (14 questions). This outcome measure was presented on a scale from 1 to a maximum of 10 points. The independent variables were intervention, gender and interaction between these variables to account for the gender-specific effects of the intervention.

### Statistical analysis

Linear mixed models were used in order to account for the dependence caused by clustering of the students into small groups. A small-group-dependent random intercept was estimated to correct for differences between the small groups that would cause correlated residuals without this procedure. A restricted maximum likelihood estimation procedure was used, and since both the number of small groups and the number of students within a small group was substantial, we used a Satterthwaite correction for the degrees of freedom. The small group was used as a random factor. Analysis was performed according to the intention-to-treat principle. After the primary analysis, a subgroup analysis was performed according to gender. Effect sizes were calculated according to Cohen’s d [22].

### Ethical considerations

Formal written permission to execute the study was obtained from the course coordinator. As this study was not subjected to a formal ethical approval process for medical education research, information about the treatment of the students is provided. This concerns the possible risks for the students, the equitability of the selection, the guarantee of privacy and confidentiality, the procedure on informed consent, and the possible safeguards to protect vulnerable populations [23,24]. In our opinion, participation in the study bore no possible risk to the students. Participation was on a voluntary basis. The students were adequately informed of the purpose of the study and their written consent was obtained. Assignment of the students to the intervention or control arm was random. The privacy of the students was guarded by the study coordinator. For the study, the examination scores were linked to a student number and the identity of the students was not disclosed. We were not aware of any vulnerable students among the participants who would have required safeguards. When developing the current study, the ethical principles of the World Medical Association Declaration of Helsinki were taken into account [25].

## Results

### Participation rate

The participation rate was 100%. Students who participated in the SGW, but did not take the formal examination, were excluded (n = 15). A total of 444 students were included in the analysis (Figure 1). There was no significant difference in the number of dropouts between the study arms.

### The effect of written questions on the formal examination score

The mean subscore on the topic tumour pathology in the formal examination per student was 7.2 (SD 1.2) in the intervention group and 6.9 (SD 1.3) in the control group (p = 0.22).

### Gender effect on formal examination score

Female students’ subscore on tumour pathology in the control group was significantly higher compared with male students: 7.1 versus 6.6 (p = 0.016). In the intervention group, the difference between female and male students was much smaller: 7.2 versus 7.1 (p = 0.55).

### Gender differences in the learning effect of the intervention of written questions

Male students in the intervention group had a 0.5 higher subscore than male students in the control group (p = 0.05); effect size was 0.35 (Table 1). In female students, the difference between the intervention and control groups was only 0.1 (p = 0.75).

**Table 1:** **Outcome measures (scale 1–10) including standard deviations and effect sizes**

	Score on tumor pathology (SD)	
Study arm	Males	Females
Intervention	7.1 (1.33)	7.2 (1.15)
Control	6.6 (1.38)	7.1 (1.24)
Effect size	0.35	0.04

## Discussion

The present randomized controlled study reveals that generation of written questions by individual (bio) medical students, followed by plenary selection of the two most relevant questions during a SGW session, seems to exert a positive learning effect on male students. The students were encouraged to formulate additional written questions on disease mechanisms. Furthermore, they were involved in a discussion to select the most relevant questions. To do this, students needed to have the ability to focus on the relevant items of the topic and express these orally to their fellow students. To our surprise, female students’ scores did not improve on this intervention. A possible explanation is a ceiling effect, as female students already performed better in comparison with their male colleagues. Another explanation could be that males were more triggered to perform better in a competitive environment of oral combat to select the best questions. Additional aspects regarding gender differences in learning will be discussed later.

### Comparison with the literature

The literature available on question asking mainly concerns observational studies using qualitative outcome measures to assess student satisfaction. To our knowledge, this is the first randomized controlled trial assessing the effect on examination scores. Bobby et al. performed a similar study among undergraduate medical students, but used a pretest–posttest design [26]. They found that formulation of questions was highly effective in understanding the topic for all students. Furthermore, Abraham undertook a similar study to ours; after a 45-minute lecture, she asked students to generate questions after discussion with their peers [27]. Similar to our study, students were asked to formulate questions and present them the following day. Students indicated that it helped them to focus on the topic, explore uncertainties and prepare for the upcoming examination. Abraham did not measure the effects on the formal examination scores, thus our study adds to Abrahams study.

Chin et al. reported that quality and type of questions posed by students determine the extent of their contribution to the construction of knowledge [28]. Basic questions, i.e. factual and procedural, generate little productive discussion, whereas wonderment questions, which are indicative of deep learning, stimulate students to hypothesize and generate explanations [28]. Students’ questions may even be used for their own examinations as Papinczak and colleagues demonstrated [29]. In their study, all first-year students were challenged to generate a bank of formative assessment questions with answers. This was found to increase students’ satisfaction and self-esteem [30]. Furthermore, it improved self-regulatory cognitive strategies [31].

The ability to generate questions is a crucial competence for active meaningful learning and it is becoming more important as modern curricula become increasingly more interactive. The increasing emphasis on dialogue (i.e. the process of questioning and answering) reflects on the new perspective of student engagement, creating a stronger partnership between students and tutors.

### Interpretation of the effects of gender

It is important to learn how male students can be challenged, as they generally perform less well than females [32-35]. Male and female students have different learning style preferences [36-38]. Female students are known to attach more importance to the principle of social constructivism in small-group settings, and confirm greater enjoyment in taking responsibility for their own learning [39]. Furthermore, there are gender differences in motivation [40]. As mentioned in the introduction, extrinsic motivation (e.g. summative tests, status and expected income) is more prominent among male students. Females students generally have a higher degree of intrinsic motivation (genuine interest in the topic). This suggests that male students need more or other challenges to motivate them to learn. To individually formulate a written question and then select the two best questions per SGW session through plenary discussion, as in our current intervention, may induce a more competitive environment in the SGW session. Males are possibly more triggered to perform better in such a learning environment, as indicated by Kilminster et al. [41]. They provided workshops on inter-professional education and found male participants and doctors were more likely to take part in role-play and tended to dominate the discussion. Further research is needed to explore other strategies to improve learning for male students.

### Implications of the study for educational practice

Implications for educational practice include the challenge to incorporate the dialogue (i.e. questioning and answering) to a greater extent in our daily teaching and learning activities. How do we make this process of asking questions the core business of a scientific curriculum? This is especially important in medical sciences education where students are trained to become academic biomedical scientists and doctors [14,42]. Does our curriculum provide enough stimuli for asking questions and pursuing the small-group dialogue? In this respect, web applications that create discussion platforms seem highly suitable. Promising results on these online platforms have already been reported [43].

### Strengths and limitations

This was a large, prospective, randomized controlled trial in a non-laboratory setting. It focused on SGW sessions in which active learning is supposed to take place. Plenary selection of the questions was based on the principle of social constructivism, which implies that knowledge construction is a shared experience [44]. Randomization was stratified for gender, study discipline and tutor, reducing the risk of bias. Both the groups had the same exposure time to SGW activity on tumor pathology. Each year, a cohort of 400 students enters our medical curriculum. In such large cohorts, it is not easy to execute an educational intervention, as the challenge of manageability can be difficult to overcome. The current intervention is suitable for large cohorts, because it is not time-consuming, as the efforts are mainly made by the students themselves. Tutors only have a facilitating role. Furthermore, the intervention seems to be generalizable to a reasonable extent as asking questions is not specific to the topic, nor to the session. It can be used in other small-group settings, such as problem-based learning sessions, team-based learning sessions and interactive lectures.

A major limitation was the fact that the intervention was small, and there is a chance that the results were coincidental, given the borderline significance (p = 0.05). Therefore, our results should be interpreted carefully, and assessment of the learning effect by replication of the study in similar settings is desirable.

Another limitation was the dual nature of the intervention, as it contains a part where individual students generate a question and formulate it in writing, and a second part consisting of prioritization of questions during a plenary discussion in the small group. It is plausible that both parts contribute to the learning effect, but, as inherent to a randomized controlled study, it is not possible to determine which factor has actually caused the effect. Bobby et al. conducted a similar study assessing the contribution of formulating questions and small group discussion separately. For high achievers, the learning effect of written questions was greater than the learning effect of the group discussion. Among low and medium achievers, the learning effect of the group discussion was greater than the learning effect of the written questions [26]. In the current study, data to stratify students into groups of high and low achievers were not available.

A final limitation concerns the outcome measure used, i.e. the score on the final examination. It was hypothesized that formulating written questions and prioritizing them would lead to a better performance. Improvement of the small-group dialogue is a likely mediating factor; however, this was not assessed systematically. Mixed-methods research, including direct observations and student surveys, would be highly suitable for this purpose.

## Conclusions

Formulating and prioritizing an extra written question during small-group work seems to exert a positive learning effect on male students. Asking students to generate written questions seems an interesting approach because it: (1) stimulates active participation and combating in debate; and (2) may stimulate student–tutor interaction. This is an interesting strategy to improve student learning as it fits well within the concept of participatory education in which students are responsible for their own learning, to a larger extent.

## Abbreviation

SGW: Small-group work
